# Neurilemmoma Masquerading a Dermoid Cyst at the Inferior Mandibular Margin: An Unusual Clinical Occurrence

**DOI:** 10.1155/crid/9976255

**Published:** 2026-03-05

**Authors:** Bibek Kattel, Akunchan Shrestha, Siddhartha Rai, Niroj Khanal, Shashi Keshwar, Mehul Rajesh Jaisani

**Affiliations:** ^1^ College of Dental Surgery, B.P. Koirala Institute of Health Sciences, Dharan, Nepal, bpkihs.edu; ^2^ Department of Oral Medicine and Radiology, B.P. Koirala Institute of Health Sciences, Dharan, Nepal, bpkihs.edu; ^3^ Department of Oral and Maxillofacial Surgery, B.P. Koirala Institute of Health Sciences, Dharan, Nepal, bpkihs.edu; ^4^ Department of Oral and Maxillofacial Surgery, Rapti Academy of Health Sciences, Dang, Nepal; ^5^ Department of Oral Pathology, B.P. Koirala Institute of Health Sciences, Dharan, Nepal, bpkihs.edu

**Keywords:** dermoid cyst, facial nerve, marginal mandibular nerve, neurilemmoma, schwannoma

## Abstract

Neurilemmomas in the submental and submandibular region represent a diagnostic challenge, often mimicking more common superficial cysts. This is a case of neurilemmoma of the marginal mandibular branch of the facial nerve, initially diagnosed as a dermoid cyst via ultrasonography in a 65‐year‐old male with an 18‐month history of a painless 1.5‐cm mass. Histopathological examination of the excised lesion confirmed neurilemmoma. The mass was encapsulated and easily detached from the surrounding tissue; thus, successful excision without postoperative neurological deficits was ensured.

## 1. Introduction

Neurilemmoma is a rare, benign, encapsulated, and slow‐growing tumor arising from Schwann cells of the nerve sheath in myelinated nerves. Approximately 25% of neurilemmomas occur in the head and neck region, where they typically present as well‐encapsulated, gradually enlarging masses attached to their nerve of origin. Although they can develop at any age, epidemiological studies indicate a peak incidence in the 2nd and 3rd decades of life [1–5]. This report presents a case of neurilemmoma in the submental region near the mandibular margin, initially misdiagnosed as a dermoid cyst. The clinical presentation, along with imaging, histopathological, and surgical findings, is described in detail.

## 2. Case Presentation

### 2.1. History and Clinical Examination

A 65‐year‐old male from Bihar, India, was referred by a dermatologist for the evaluation of swelling on the left side of the chin persisting for 18 months. The lesion was insidious in onset, slowly progressing and stabilizing at its maximum size at around 9 months, then stopped growing since the last 8–10 months. His past medical history and family history were unremarkable. However, he had notable habits of chewing tobacco for 5 years and betel quid consumption for over 40 years. There was no history of trauma to the orofacial region, infection, deranged function of the TMJ, and no difficulty in opening and closing the jaw. The patient denied tooth pain, fever, weight loss, night sweating, tingling, and burning sensation over the chin and lower lip.

Intraoral examination was within normal limits with no relevant findings related to the lesion. Extraoral examination revealed a single‐localized, well‐defined, unilateral, oval‐shaped sessile mass (Figure 1) of size 1–1.5 cm in dimension, present on submental region about 1 cm inferior to inferior border of mandible on the left parasymphysis region. The swelling was soft to firm in consistency with normal overlying skin with no secondary changes, nontender, nonreducible, noncompressible, and nonpulsatile, with no signs of inflammation; cervical lymph nodes were within normal limits. The extension of the lesion could be palpated beneath the growth up to 1–1.5 cm deep. No neurological abnormality was noted. All the teeth responded positively to pulp testing. The remainder of the physical examination, including head and neck, was within normal limits.

Figure 1(a) Extraoral photograph of the patient showing oval‐shaped single sessile mass over the left mandibular parasymphysis region. (b) Close‐up view focusing on the oral region, providing context for the lesion′s location. (c) Detailed close‐up of the lesion, highlighting its morphological features.

**Figure:**
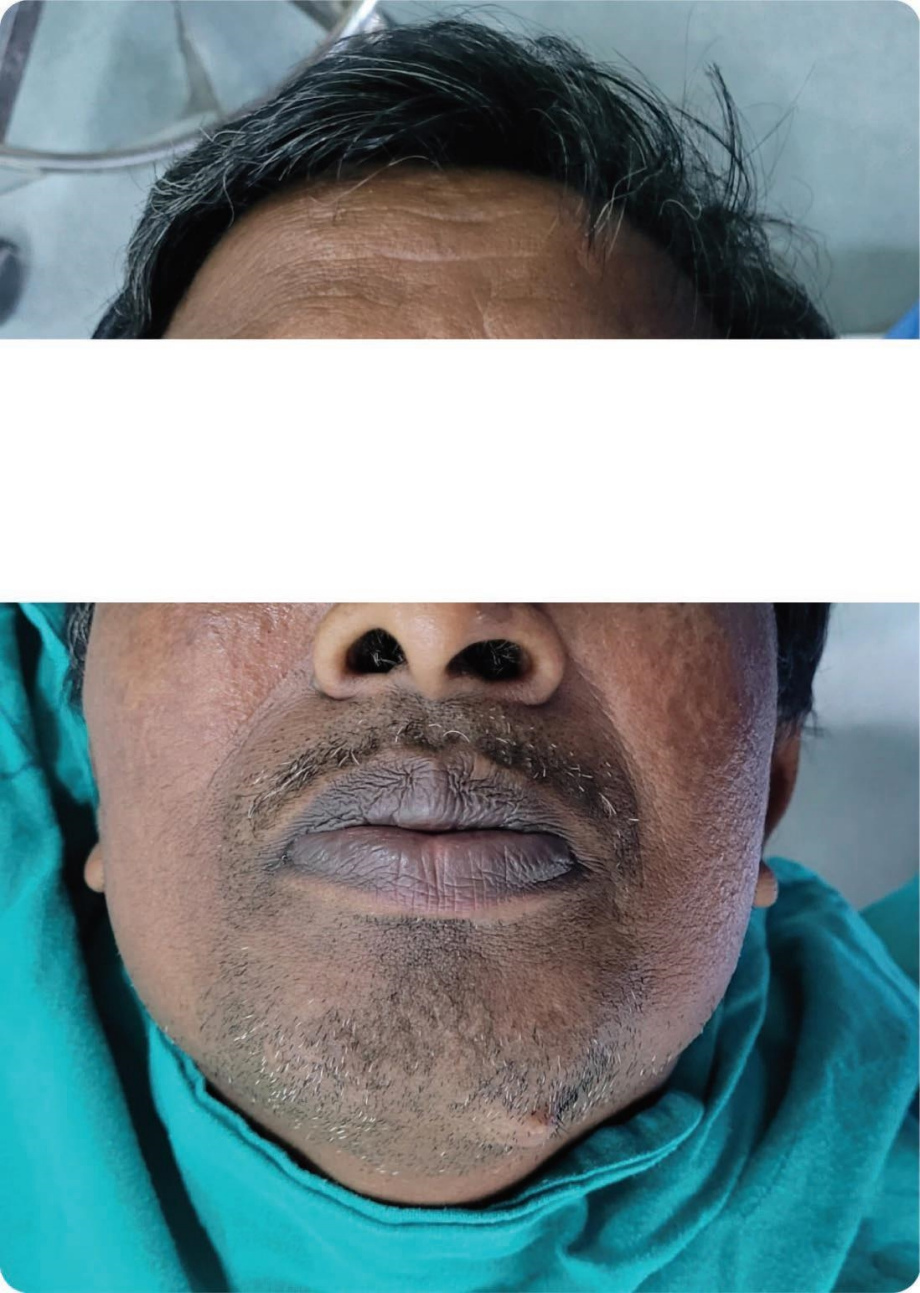
(a)

**Figure:**
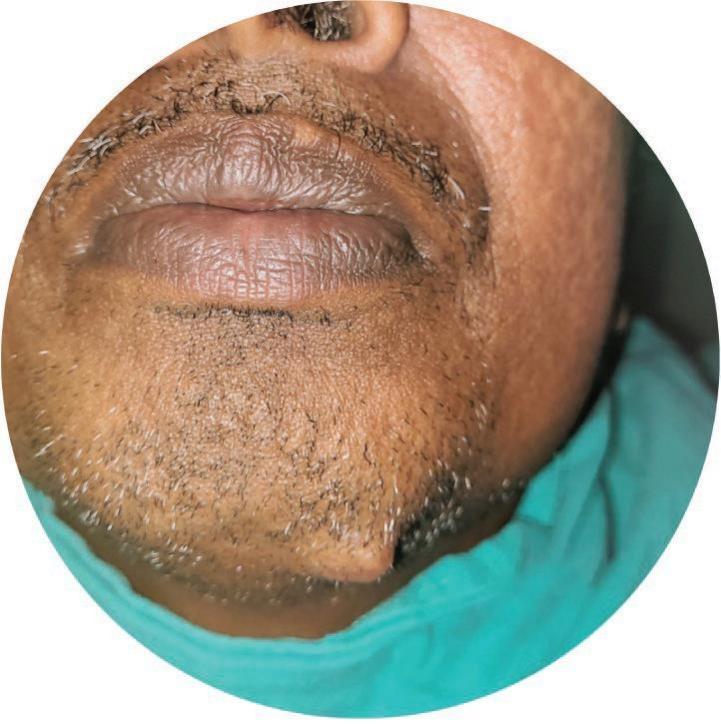
(b)

**Figure:**
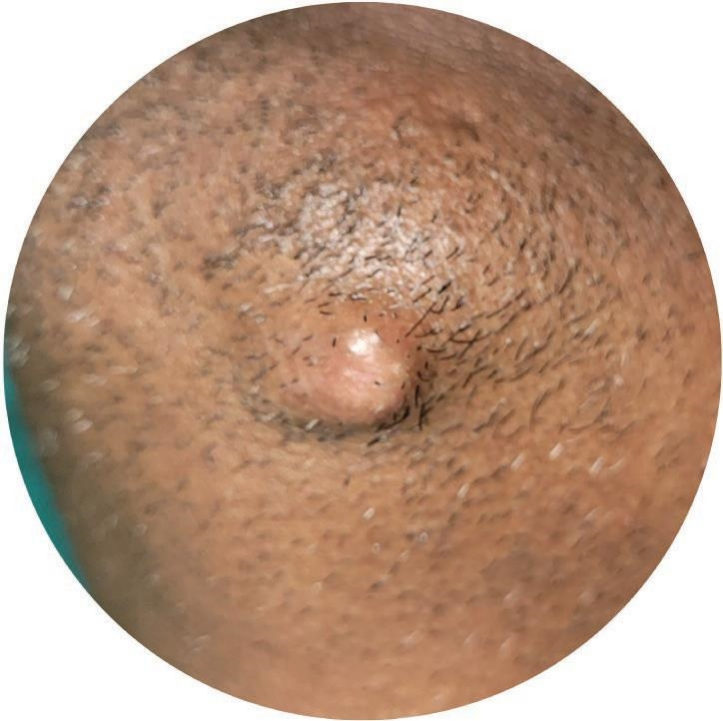
(c)

### 2.2. Investigations

**Figure 2:**
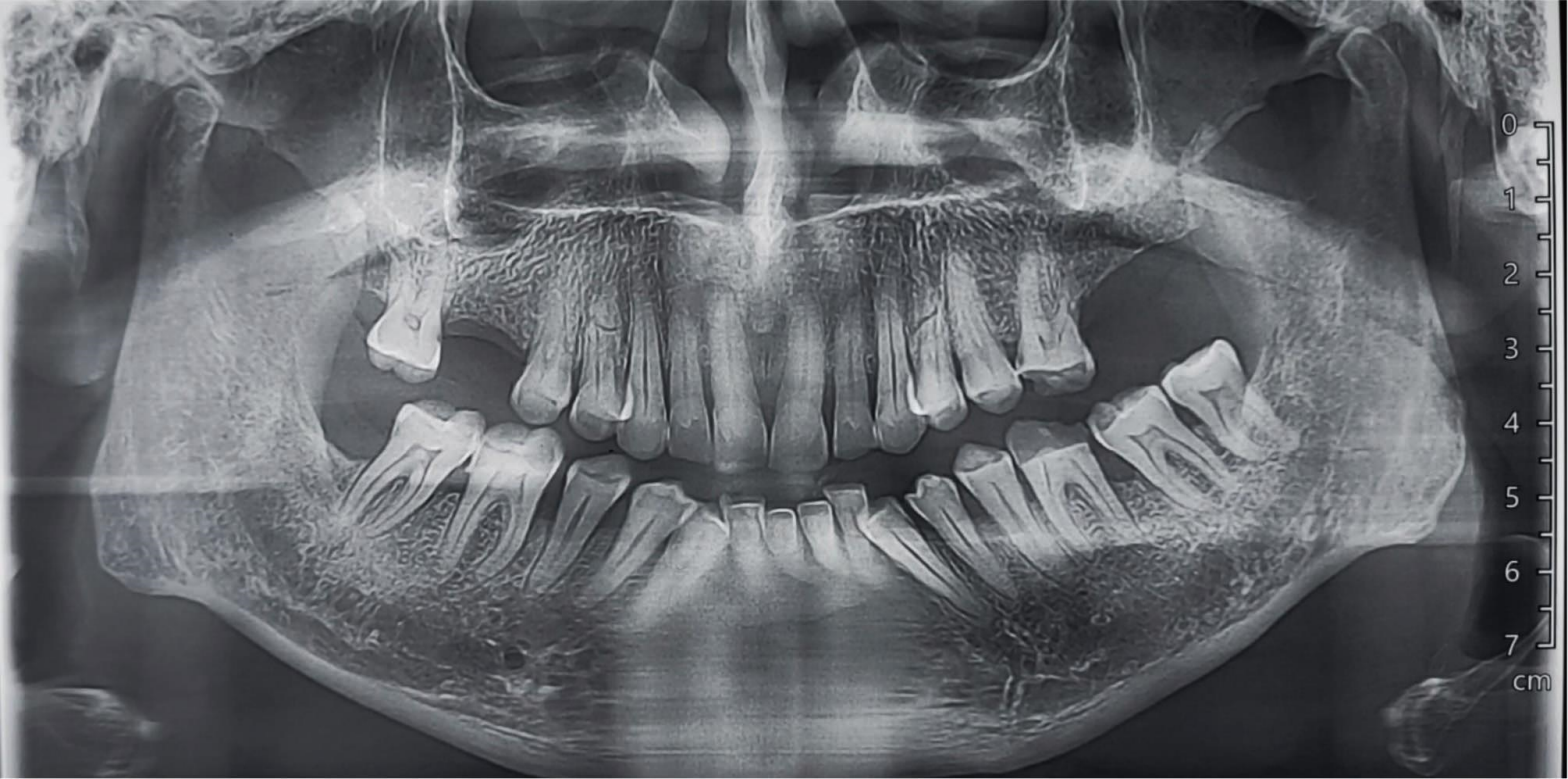
Orthopantomogram (Failed to disclose any abnormalities).

Hemogram was within normal limits. Orthopantomogram (Figure 2) failed to disclose any abnormalities. Ultrasonography (USG) revealed a well‐defined, ovoid, heterogeneous hypoechoic lesion measuring approximately 16.2 × 7.4 *m* *m*, located in the subcutaneous plane abutting the skin. (Figure 3a,b) There were no internal vascular signals on Doppler evaluation, and no evidence of calcification or cystic degeneration. Posterior acoustic enhancement was noted. A linear ultrasound probe (5–10 MHz) was used for the scan.

Figure 3Ultrasonography of the anterior chin lesion. (a) Well‐defined mixed echoic lesion in the subcutaneous plane. (b) Measurement of the lesion (16.22 × 7.49 *m* *m*).

**Figure:**
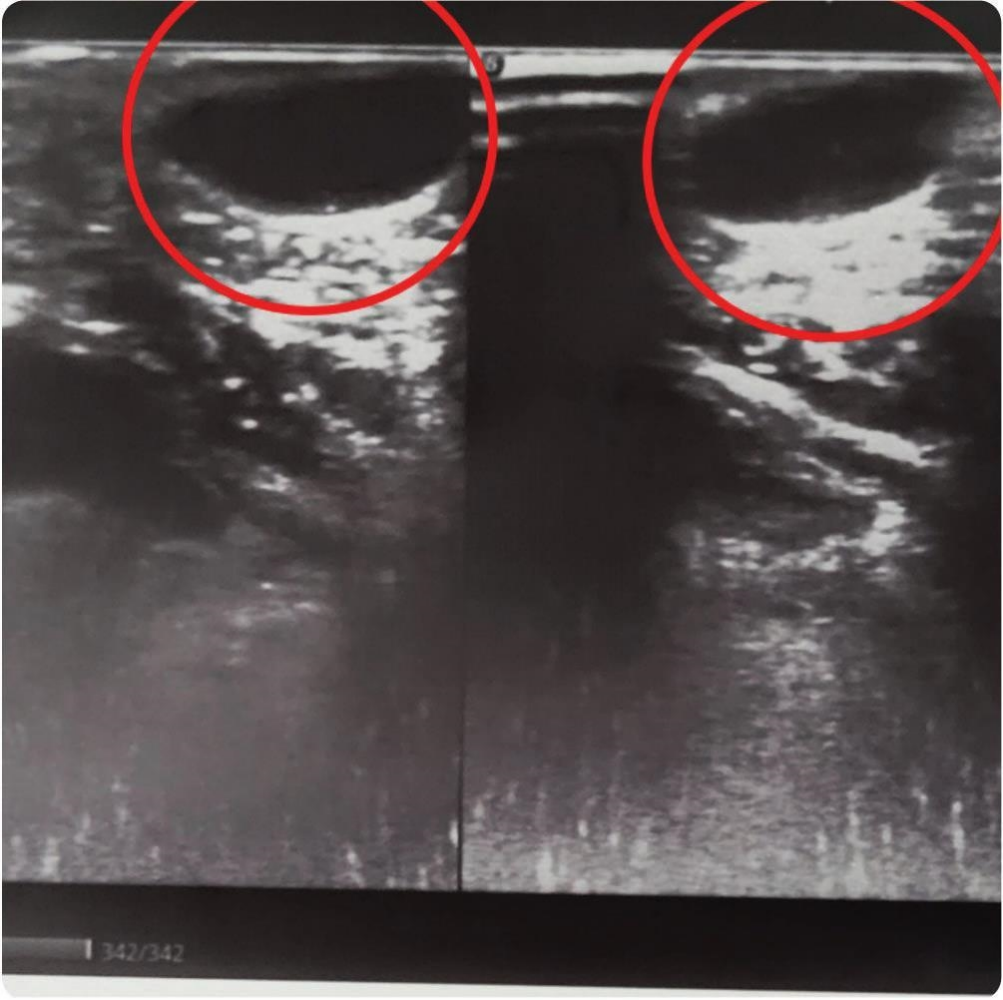
(a)

**Figure:**
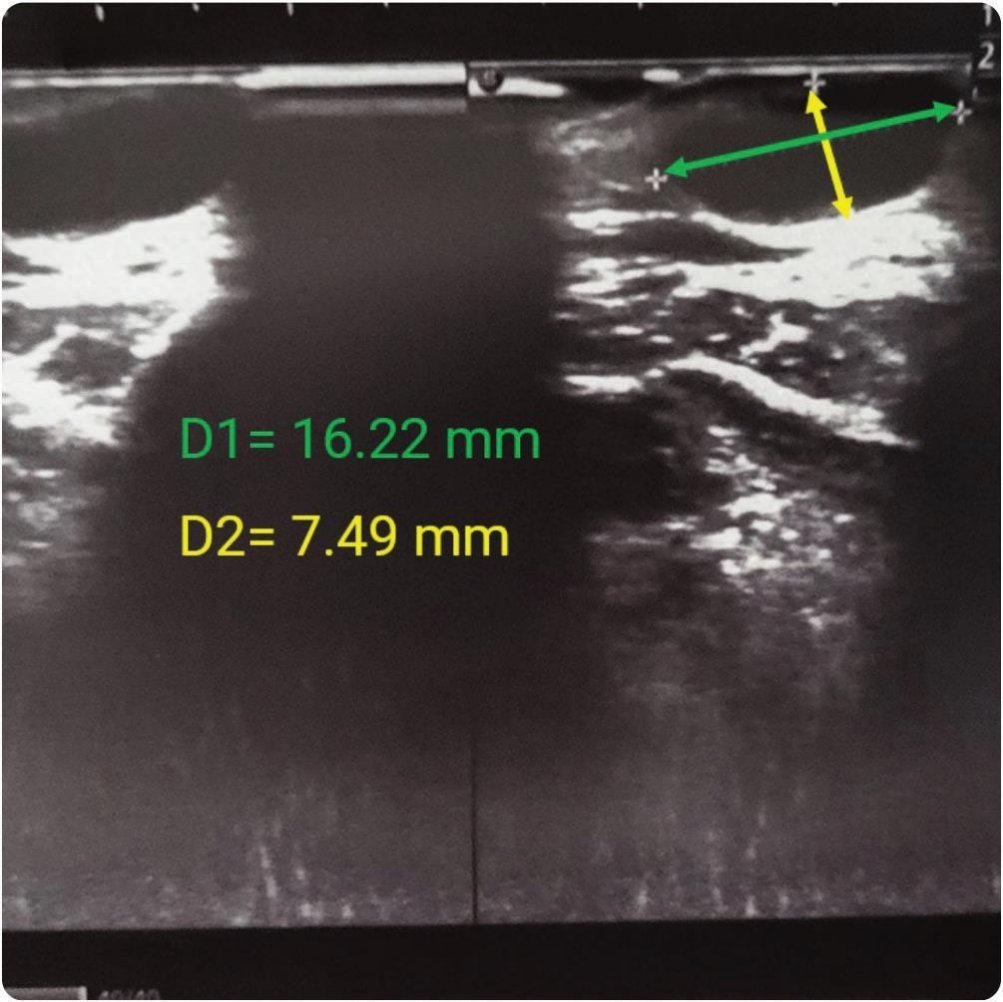
(b)

A dermoid/epidermoid cyst was initially favored due to its superficial location, absence of internal vascularity, and the presence of posterior acoustic enhancement, which are the features that are typically suggestive of a cystic lesion despite a relatively solid appearance. However, a definitive diagnosis could only be established through histopathological examination. Magnetic resonance imaging (MRI) was not performed due to the patient′s financial constraints.

On aspiration, the lesion was found to be solid, prompting the decision for excisional biopsy.

### 2.3. Treatment

The patient underwent surgical excision of the mass under local infiltration with 3 mL of 2% lignocaine with adrenaline in a concentration of 1:200000, after taking written consent. After a surgical skin incision was made directly over the swelling, blunt dissection was done carefully to expose the lesion. The mass was found to be encapsulated and attached to the small peripheral nerve. Further, blunt dissection and complete removal was accomplished by transecting the attachment to the nerve. Hemostasis was achieved, and the surgical field was thoroughly irrigated. The incision was closed in layers with absorbable sutures for the deeper tissues and nonabsorbable sutures for the skin.

### 2.4. Gross and Histopathological Examination

**Figure 4:**
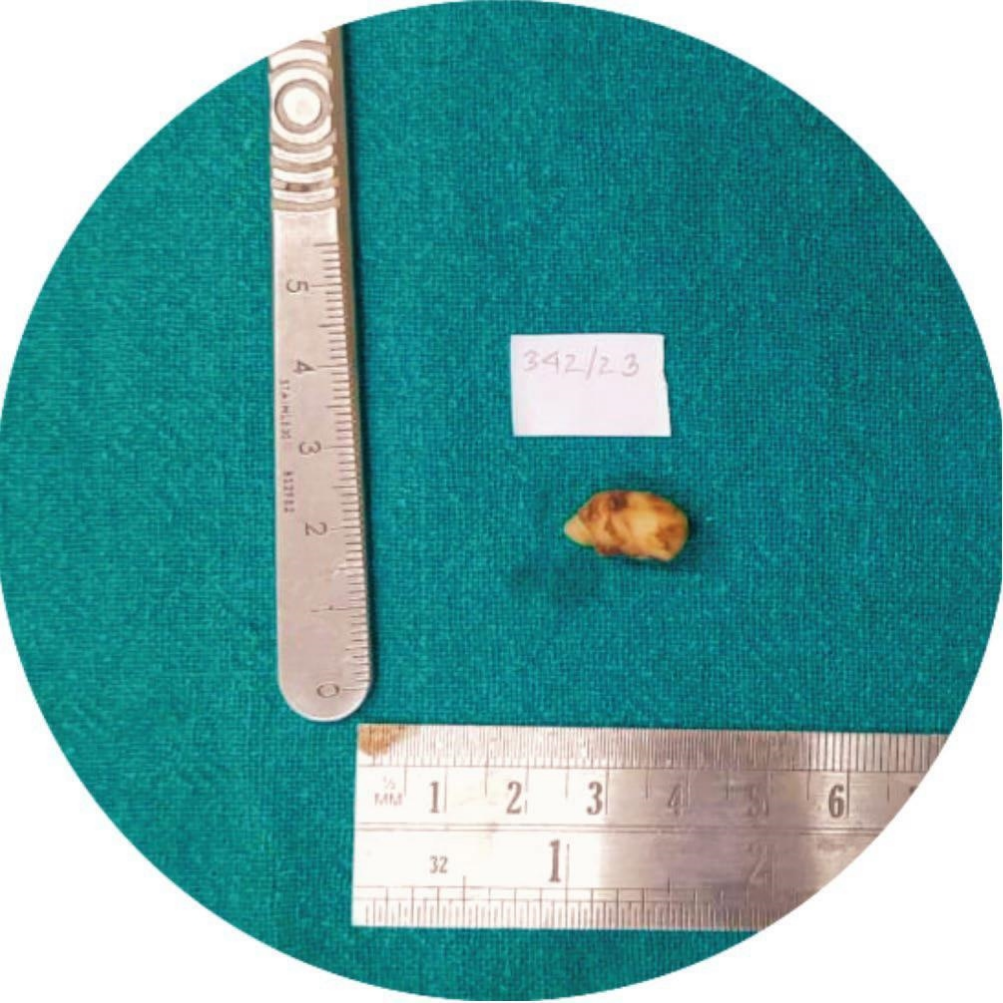
Excised tissue.

The excised tissue was single, firm, white to light yellow in color measuring 17 × 5 × 8 *m* *m* ^3^ of the largest dimension (Figure 4). Histopathological examination revealed the presence of fibrocellular connective tissue stroma with streaming fascicles of spindle‐shaped Antoni A and randomly‐arranged Antoni B cells, accompanied by a central acellular eosinophilic area of Verocay bodies (Figure 5a,b). Correlating clinically, the histopathological findings are suggestive of neurilemmoma.

Figure 5(a) Numerous endothelial cells lined blood vessels of variable sizes along with inflammatory cells infiltrate predominantly lymphocytes. Periphery of the specimen is lined by a fibrous capsule and adipocytes. (b) The photomicrograph at 10× magnification shows Antoni A and Verocay body (arrow), Antoni B (∗).

**Figure:**
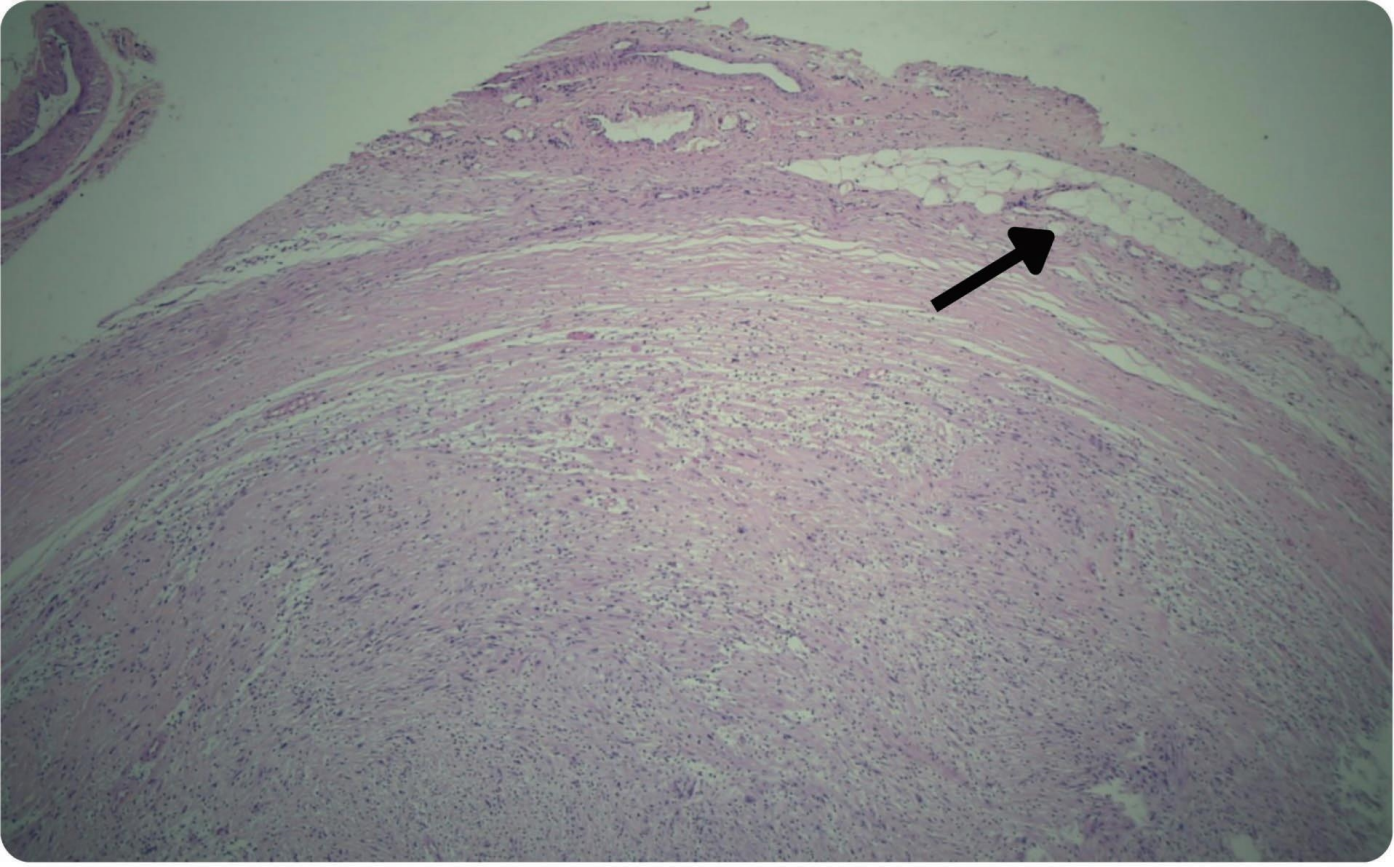
(a)

**Figure:**
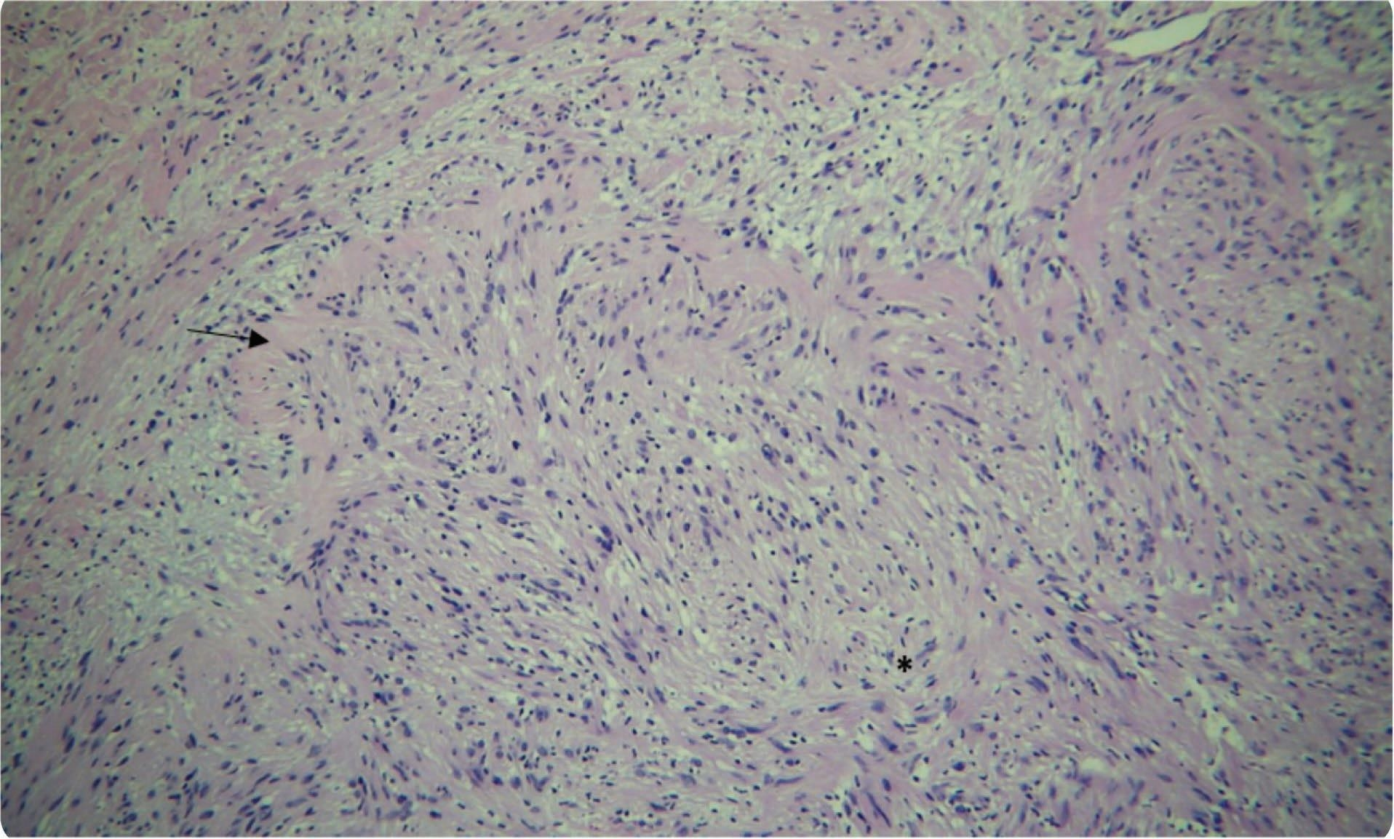
(b)

### 2.5. Outcome and Follow‐Up

The patient was prescribed amoxicillin 500 mg three times a day for 3 days, ibuprofen 400 mg three times for a day as needed, and advised to maintain the wound hygiene and follow‐up after 7 days for wound assessment. Postoperative recovery was uneventful. Regarding neurological assessment, postoperative follow‐up included motor evaluation (symmetry of lower lip movement during smiling, puckering, and depression).

The patient showed no motor weakness of the marginal mandibular branch in the lower lip and chin region. Follow‐up was carried out at postoperative Day 7. No neurological complications were observed.

## 3. Discussion

Neurilemmoma typically presents with a smooth and uniform gross appearance and is often encased by or attached to the nerve of origin. Although more commonly found in the extremities, approximately 25%–45% of cases originate from neural structures in the head and neck region, with rare occurrence in the maxillary region [6, 7]. Among these, 20% occur intraorally, with the tongue being the most common intraoral site, followed by the palate, floor of the mouth, buccal mucosa, gingiva, lips, and vestibular mucosa [8, 9].

Typically, a neurilemmoma is attached to its nerve of origin, grows slowly, and is generally painless. When occurring along peripheral nerves, diagnosis can be facilitated through clinical evaluation and imaging studies. Neurilemmomas are usually firm, round, and freely movable from surrounding soft tissues. In early stages, they are often asymptomatic, presenting only as a palpable lump, while pain or neurological symptoms may arise due to mass effect on adjacent structures [3, 4].

Although these tumors primarily present in the 2nd or 3rd decade of life, they can develop at any age, with no clear sex predilection. They typically range from 0.5 to 3 cm in size, rarely exceeding 5 cm. Neurilemmoma can affect individuals of all age groups but is most commonly found in the 2nd and 3rd decades of life. [5, 8, 9] The rarity of neurilemmomas, combined with their diverse morphological and radiological presentations, poses a challenge in differential diagnosis. Head and neck neurilemmomas are frequently misdiagnosed, as preoperative investigations were often inconclusive [10, 11].

A definitive diagnosis is established through histopathological examination [12]. In the present case, limited literature is available on lesions occurring at this specific site, as neurilemmomas predominantly arise near the mental foramen or within bone, primarily attached to major nerves rather than peripherally. Consequently, it was misdiagnosed as a dermoid cyst. Therefore, the lesion was excised for biopsy after obtaining informed consent from the patient regarding potential consequences. The biopsy confirmed the definitive diagnosis of neurilemmoma.

Microscopic evaluation of neurilemmomas shows two distinct histopathological patterns. The Antoni A pattern has densely packed spindle cells arranged in a palisading fashion around acellular, eosinophilic Verocay bodies. In contrast, the Antoni B pattern features a loose, hypocellular arrangement adjacent to Antoni A. Immunohistochemical staining for S‐100 protein, a neural crest marker, is a key diagnostic feature [5, 12, 13]. Various diagnostic modalities were discussed with the patient; however, due to financial constraints, he was unable to afford them. The definitive diagnosis was established based on histopathological examination. Previous reports of subcutaneous neurilemmomas of the face have not identified the specific nerve of origin. Documented cases include tumors in the upper lip, ala nasalis, and areas lateral to the oral commissure and mental foramen [14–16]. In two cases [15, 16], the mental nerve was preserved during tumor resection, resulting in sensory recovery, likely due to the tumor being separated from the nerve. However, these tumors originated from the mandibular nerve within the oral cavity rather than the mental foramen. Notably, to the best of our knowledge, only one previous case of neurilemmoma involving a branch of the peripheral facial nerve has been reported [14]. While Although subcutaneous neurilemmomas typically do not present with neurological symptoms, intraoperative nerve damage may lead to postoperative deficits [14]. In this case, no sensory deficit was observed as the tumor originated from the peripheral portion of the marginal mandibular branch.

## Funding

No funding was received for this manuscript.

## Ethics Statement

The authors have nothing to report.

## Consent

Written informed consent was obtained from the patient to publish this report in accordance with the journal′s patient consent policy.

## Conflicts of Interest

The authors declare no conflicts of interest.

## Data Availability

The data that support the findings of this study are available on request from the corresponding author. The data are not publicly available due to privacy or ethical restrictions.
